# Poor Cognitive Flexibility in Eating Disorders: Examining the Evidence using the Wisconsin Card Sorting Task

**DOI:** 10.1371/journal.pone.0028331

**Published:** 2012-01-12

**Authors:** Kate Tchanturia, Helen Davies, Marion Roberts, Amy Harrison, Michiko Nakazato, Ulrike Schmidt, Janet Treasure, Robin Morris

**Affiliations:** 1 Division of Psychological Medicine, Institute of Psychiatry, King's College London (KCL), London, United Kingdom; 2 Department of Psychology, Institute of Psychiatry King's College London (KCL), London, United Kingdom; University of Granada, Spain

## Abstract

**Background:**

People with eating disorders (ED) frequently present with inflexible behaviours, including eating related issues which contribute to the maintenance of the illness. Small scale studies point to difficulties with cognitive set-shifting as a basis. Using larger scale studies will lend robustness to these data.

**Methodology/Principal Findings:**

542 participants were included in the dataset as follows: Anorexia Nervosa (AN) n = 171; Bulimia Nervosa (BN) n = 82; Recovered AN n = 90; Healthy controls (HC): n = 199. All completed the Wisconsin Card Sorting Task (WCST), an assessment that integrates multiple measurement of several executive processes concerned with problem solving and cognitive flexibility. The AN and BN groups performed poorly in most domains of the WCST. Recovered AN participants showed a better performance than currently ill participants; however, the number of preservative errors was higher than for HC participants.

**Conclusions/Significance:**

There is a growing interest in the diagnostic and treatment implications of cognitive flexibility in eating disorders. This large dataset supports previous smaller scale studies and a systematic review which indicate poor cognitive flexibility in people with ED.

## Introduction

There is a growing literature in the neurobiological underpinnings of the psychopathology of eating disorders (ED) [1]. A salient feature is that people with ED frequently present with inflexible behaviours around eating related issues (e.g. counting calories, exercising), have rigid rituals around the daily routine (e.g. cleaning, housekeeping, homework) and experience difficulties in seeing alternative ways of coping with problems. Consistent with this, difficulties with cognitive flexibility have been shown to be an important risk and maintenance factor, for example, in anorexia nervosa (AN) [2], [3]. The neurobiological basis for this impairment is not established, but some evidence from studies assessing first-degree relatives suggests that it could be a candidate endophenotype [3]–[5]. Recently, this trait has been found in adolescent cases of people with AN [6] and in small-scale studies with people who have recovered from AN [5], [7].

In this context, the assessment of cognitive flexibility using standardised tests to measure set-shifting and problem solving is relevant to clinical practice. One commonly used neuropsychological measure of cognitive flexibility (or set-shifting ability) is the Wisconsin Card Sorting Task (WCST [8]). This procedure integrates multiple measurements of executive processes and is one of the most widely reported neuropsychological tasks, despite some acknowledged weaknesses in interpretation of the profiles (e.g. difficulties in task performance could be caused by set shifting, poor abstraction and conceptualization, or attentional problems). One of the main outcomes of the WCST is the measurement of perseveration; defined as repetitive responses to a stimulus/rule that continues despite a shift in the stimulus requiring a different response.

A systematic review of set-shifting in ED included five experimental studies using the WCST [9] based on all available studies before December 2005. It analysed the performance of 73 patients diagnosed with anorexia nervosa (AN) compared to 80 healthy controls (HC), the results suggesting that AN patients have prominent difficulties in set-shifting. Subsequent studies have confirmed these findings [4], [5], [10]. However, there remains limited data available for other categories of ED, such as bulimia nervosa (BN) [11].

This brief report explores WCST performance and various other clinical outcomes in a substantially large dataset collated from several published studies that have used this task in accumulated samples of people with a lifetime history of an ED (AN and BN) and people who have recovered from an ED (RecAN), with HC comparisons.

## Methods

### Participants

All participants were recruited between 2006 and 2011 in our department (n = 542 in total). In line with the ethical standards laid down in the 1964 Declaration of Helsinki, all studies had received approval from the ethical committee of the South London and Maudsley (SLaM) NHS Foundation Trust. All participants provided written informed consent prior to their inclusion in the study. ED patients were recruited from the SLaM Eating Disorders inpatient or outpatient units and had been diagnosed by experienced ED clinicians as fulfilling DSM-IV criteria for AN or BN. HC and recAN participants were recruited via advertisements in the local community, and through circular emails sent around to King's College London students and staff. The inclusion and exclusion criteria for the recAN group was based on Bardone-Cone et al. 2010, who state the definition of this group should include physical, behavioural, and psychological components, such that recovered participants were included if they: a) had a body mass index BMI (weight/height^2^) above 18.5, b) had restored menstruation for at least a year prior to recruitment; and c) had an absence of ED behaviours such as restriction or binge-purge symptoms during this one year period. HC participants were excluded if they had any history of EDs, head injury or psychiatric illness. All participants were female and aged between 18 and 55 years old.

Cases from the final dataset were excluded if age, current BMI, length of illness or WCST original data were missing. Additionally, ED cases were excluded if BMI>25, and HCs were excluded if BMI<19. Of those with AN, 8 cases were excluded because their BMI was above the diagnostic cut off point (>18). From the patient sample, BN participants were outpatients or from a community sample (n = 82); AN participants were inpatients (n = 90), outpatients and from a community sample (n = 81). Table 1.

**Table 1:** Clinical and Demographic Characteristics of the Healthy Control (HC), Eating Disorder (AN, BN) and Recovered AN (REC AN) Groups.

	HC n = 199	AN n = 171	BN n = 82	Rec AN n = 90	Test Statistics
**Age**	27.7 (8.8)	25.4 (8.2)	27.3 (8.3)	30.7 (11.1)	F(3,541) = 7.092 p<0.001
**BMI**	21.9 (1.9)	15.2 (1.9)	21.3 (2.4)	20.5 (1.6)	F(3,540) = 375.408 p<0.001

BMI = Body mass index (weight/height^2^).; HC = Healthy control participants; AN = Anorexia nervosa participants; BN = Bulimia nervosa participants; RecAN = Recovered anorexia nervosa participants. Test statistics are ANOVAs and descriptive statistics are means followed by standard deviations in parentheses.

### Measures

The Wisconsin Card Sorting Test [8] (WCST Computerised version 4: CV4) was used in all the studies, presenting the task graphically on a computer screen. The WCST entails matching stimulus cards with one of four category cards, in which the stimuli are multidimensional according to colour (C), shape (S) and number (N), each dimension defining a sorting rule. By trial and error, the participant has to settle a preordained sorting rule given just the feedback (“Right” or “Wrong”) on the screen after each sort. After 10 consecutive correct sorts the rule changes. There are up to six attempts to derive a rule, providing five rule shifts in the following sequence (C-S-N-C-S-N), with each rule attainment referred to as ‘completing a category.’ Participants are not informed of the correct sorting principle and that the sorting principal shifts during the test; Testing continues until all 128 cards are sorted and irrespective of whether the participant achieves completes all the rule shifts. Two types of errors are possible, perseverative errors, in which the participant makes a response in which they persist with a wrong sorting rule, and non-perseverative errors.

Many studies using this test present two or three scores as indices of performance, but given the relatively large sample size, this was increased to 11 principal measures grouped into four main types, as follows: A) *General Performance measures*: The number of trials administered, the total correct responses, the total response errors and the number of categories completed; B) *Perseveration*: Perseverative responses (any response that fitted the criteria for perservation), perseverative errors (only perseverative responses that are also errors) and non-perseverative errors; C) *Conceptual Ability*. The number of trials needed to complete the first category and percentage of conceptual level responses; and D) *Response Consistency*: This includes a failure to maintain set measure, computed as the number of times the participant makes between 5–9 correct responses in a row, reflecting efficiency during the test; and learning to learn, a measure of decrement in the number of responses needed to achieve each successive category.

For the number of trials to complete the first category measure, a score of 128 was given if no category was achieved. As shown in Table 2, percentages of responses or errors were calculated where appropriate by dividing the scores by the total number of trials administered.

**Table 2:** Wisconsin Card Sort Task Data (Means, Standard Deviations and Effect Sizes) for the Healthy Control, Anorexia Nervosa, Bulimia Nervosa and Recovered Anorexia Nervosa Groups.

	HC = 199	AN = 171	BN = 82	ANrec = 90	Test Statistics
**General performance measures**					
Number of trials administered	88.3 (16.7)	101.4 (21.6)* **0.7**	102.8 (19.7)* **0.8**	91.7 (19.5) 0.1	F(3,541) = 19.5,p<0.001
Total correct responses	70.2 (7.8)	71.2 (11.4) 0.1	70.2 (12.3) 0	69.9 (8.0) 0	F(3,541) = .525 p = .66
Total response errors (%)	18.3 (10.5)	29.9 (20.6)* **0.7**	30.5 (21.6)* **0.8**	22.7 (17.5) 0.3	F(3,541) = 17.6 p<0.001
	18.8 (8.4)	27.3 (15.9)* **0.6**	29.4 (16.4)* **0.9**	21.2 (12.4) 0.2	F(3,541) = 19.634 p<0.001
Total categories completed	5.9 (0.4)	5.1 (1.6)* **0.7**	5.1 (1.4)* **0.9**	5.5 (1.0) **0.6**	F(3,459) = 12.744 p<0.001
**Perseveration**					
Perseverative Responses (%)	10.0 (6.6)	16.9 (14.4)* **0.6**	18.9 (17.1)* **0.8**	13.0 (12.7) 0.3	F(3,541) = 14.586 p<.001
	10.2 (5.1)	15.5 (11.8)* **0.6**	15.6 (10.8)* **0.7**	12.2 (9.6) 0.2	F(3,540) = 12.468 p<0.001
Perseverative errors (%)	8.3 (3.8)	15.1 (11.9)* **0.8**	15.6 (12.7)* **0.9**	11.6 (10.2) **0.5**	F(3,541) = 19.144 p<0.001
	9.4 (4.2)	13.9 (9.6)* **0.6**	14.3 (9.7)* **0.7**	11.1 (7.5) 0.3	F(3,541) = 13.799 p<0.001
Non-perseverative errors (%)	9.2 (5.9)	14.7 (11.4)* **0.6**	15.0 (11.1)* **0.7**	10.4 (7.8) 0.1	F(3,536) = 14.997 p<0.001
	9.4 (5.0)	13.3 (8.6)* **0.5**	13.6 (8.0)* **0.7**	10.0 (6.3) 0.1	F(3,541) = 13.500 p<0.001
**Conceptual Ability**					
Trials to complete first category	13.1 (6.5)	18.7 (19.1)* **0.4**	17.3 (17.0) 0.3	14.2 (7.4) 0.1	F(3,462) = 4.910 p<0.001
Conceptual level responses (%)	66.2 (6.5)	62.9 (15.7) 0.2	60.4 (19.0)* **0.5**	64.2 (10.1) 0.2	F(3,539) = 4.575 p<0.001
	75.7 (10.4)	64.7 (20.3) **0.7**	63.5 (21.1)* **0.8**	70.0 (19.5) **0.4**	F(3,539) = 13.767 p<0.001
**Response Consistency**					
Failure to maintain set	0.4 (0.8)	0.7 (1.1) 0.3	3.0 (13.0)* 0.3	0.5 (1.0) 0.1	F(3,463) = 4.508 p<0.01
Learning to learn	−.2 (2.4)	−1.2 (6.3) 0.2	−2.7 (9.5)* **0.4**	−1.5 (5.8) 0.3	F(3,443) = 3.339 p<0.01

HC = healthy control group; AN = anorexia nervosa group; BN = bulimia nervosa group; Rec AN = recovered AN group. Means and standard deviations are reported followed by effect sizes (moderate to large effect sizes highlighted in bold). Where percentages are shown (%), the total scores (responses or errors) are divided by the total number of trials in the test.

*indicate significant difference from HC group. Test statistics are based on ANOVAS.

### Statistical Analysis

The data were inspected using histograms and Kolmogorov-Smirnov tests to assess assumptions of normal distribution. A one-way ANOVA was applied to analyse between group differences for each measure. Alpha was set at *p*<0.05 unless Bonferroni's correction for multiple comparisons was applied as indicated below. Cohen's *d* (mean_1_-mean_2_/pooled standard deviation) was calculated to provide effect sizes for normally distributed data, with an effect size of <0.2 defined as small, <0.5 defined as medium and >0.8 defined as large [12].

## Results

### Clinical and Demographic Data

Table 1 below provides clinical and demographic information for the participant groups. All groups were well matched for age; however, the recAN group was significantly older than the other groups. As expected, there was a significant main effect of group for participants' BMI, with the AN group having a significantly lower BMI than other groups (p≤0.001).

Table 2 compares between group performances on the WCST. All domains showed significant differences and moderate effect sizes between groups, except WCST total correct and failure to maintain set. AN and BN groups performed significantly worse compared to HC in the majority of the task aspects. The BN group showed poorer performance in areas of: 1) Conceptual level of responses (reflecting insight into the correct sorting principle, i.e. 3 correct responses in a row usually would not occur by chance alone); 2) WCST Learning to Learn (this score can be calculated only for patients who complete 3 or more categories/stages of the test); The AN group took significantly more trials to complete the first category than HC group. The recovered AN group performed significantly worse than HCs in the domain of perseverative errors, but significantly better than AN group.

An additional analysis was conducted to observe if inpatient and outpatients with AN were different in WCST performance, with the possibility that inpatients would be more severely affected. Table 3 also shows the variables, and their respective effect sizes between IP and OP, The outpatient and community sample had significantly better performance in comparison to inpatients with AN in non perseverative errors p<0.05 and categories completed p<0.05.

**Table 3:** Wisconsin Card Sort Task Data (Means, Standard Deviations and Effect Sizes) for the Healthy Control, AN Inpatient (IP) and AN Outpatients (OP).

	HC = 199	AN IP = 87	ES AN IP/OP	AN OP = 81
Number of trials administered	88.3 (16.7)	105.7 (21.8) **0.9**	d = **0.4**	96.8 (20.6) **0.5**
Total correct responses	70.2 (7.8)	71.1 (13.5)		71.7 (8.7)
Total response errors (%)	18.3 (10.5)	34.1 (22.9) **1.3**	d = **0.4**	25.2 (16.4) **0.5**
	18.8 (8.4)	30.5 (18.0) **1.0**	d = **0.4**	23.9 (12.3) **0.5**
Total categories completed	5.9 (0.4)	4.7 (1.9) **1.1**	d = **0.5**	5.5 (1.2) **0.6**
Perseverative responses (%)	10.0 (6.6)	19.0 (16.6) **0.8**	d = 0.3	14.7 (11.5) **0.6**
	10.2 (5.1)	17.0 (14.1) **0.8**	d = 0.3	13.8 (8.6) **0.6**
Perseverative errors (%)	8.3 (3.8)	17.0 (13.5) **1.0**	d = 0.3	13.2 (9.7) **0.8**
	9.4 (4.2)	15.2 (11.4) **0.8**	d = 0.3	12.5 (7.2) **0.6**
Non-perseverative errors (%)	9.2 (5.9)	17.0 (12.9) **0.9**	d = **0.5**	11.9 (8.1) **0.4**
	9.4 (5.0)	15.1 (9.6) **0.8**	d = **0.5**	11.3 (6.5) **0.3**
Trials to complete first category	13.1 (6.5)	20.6 (21.5) **0.6**	d = 0.2	16.3 (15.7) 0.3
Conceptual level responses (%)	66.2 (6.5)	61.4 (18.4) **0.4**	d = 0.2	64.7 (12.1) 0.2
	75.7 (10.4)	60.5 (22.7) **1.0**	d = **0.4**	69.1 (17.3) **0.5**
Failure to maintain set	0.4 (0.8)	0.9 (1.1) **0.5**	d = 0.3	0.6 (1.0) **0.2**
Learning to learn	−.2 (2.4)	−1.7 (7.5) 0.3	d = 0.1	−.8 (5.1) 0.1

Means, standard deviations and effect sizes (ES) between the HC and the AN sub-group. Moderate to large effect sizes (ES) highlighted in bold.

## Discussion

This study explored WCST performance as a measure of cognitive flexibility, reporting various outcomes in a large dataset of actively ill, recovered and healthy controls collated from several studies conducted within our department. Patients with AN and BN performed poorly in almost all domains of WCST. Results showed that in terms of flexibility (perseverative errors), there was no significant difference between inpatient and outpatients with anorexia, but rather inpatients made significantly more nonperseverative errors, a finding which can reflect problems with attention and poor nutritional status. People with a past history of AN showed better performance than actively ill participants; however perseverative errors, conceptual level responses, and number of categories completed (the main flexibility outcomes) were significantly impaired compared to HC participants. The effect sizes, however, were smaller between HC and people with a past history of AN suggesting that flexibility can be improved relative to active illness state.

In general, our results clearly replicate studies which report poor cognitive flexibility in AN [4], [13], [14]. This study extends knowledge about set-shifting in BN, showing that patients with BN perform as poorly on the WCST as those with AN. People in a stage of good recovery from AN still had problems with perseverations and conceptual strategy use in this task. A recent report on a similar large dataset using a different measure of set-shifting – the Brixton Special anticipation task [15] - found that patients with AN performed significantly worse than HC and BN participant groups. In contrast, in the current study, we found that both actively ill ED patient groups (AN and BN) had worse performance in comparison to HCs and in some aspects of the tasks (e.g. perseverative responses and conceptual level of responses), people who had recovered also showed poor performance. One possible explanation for this finding could be that although both the Brixton task and WCST measure flexibility, they differ in terms of their complexity. In the Brixton task, participants are told explicitly that the sorting principle will change and are therefore alert to future rule changes. In the WCST, participants must identify the rule in order to respond correctly, with this rule subject to modification. Thus, the WCST may involve increased levels of ambiguity, as unlike the Brixton task, participants are not explicitly told that the rules will change throughout the task. The WCST is dependent on cognitive operations such as searching for a new category and consolidating the correct classification category. The tasks also differ in how feedback is provided. Whereas the WCST provides feedback in which participants are told they are “right” or “wrong,” for the Brixton task, the designated correct answer is provided by definition when the next array is shown. This type of feedback is arguably less pronounced and requires the additional process of remembering the immediately previous response and matching for correctness. In summary, the instructions are more explicit in the Brixton task and feedback is arguably more pronounced in the WCST.

Therefore an explanation for this finding could be that patients with BN failed to learn from the feedback on WCST but did well in a relatively simple switching task. The current study shows that people with ED were not able to learn from the feedback as efficiently as HCs. The recovered AN group showed intermediate scores between AN and HC for the Brixton task, and their performance was not significantly different from HCs. In the WCST, people who were recovered demonstrated a significantly higher number of perseverative errors compared to HC and showed difficulties in performing strategically on the WCST, suggesting that with more complex tasks, they performed poorly. This finding supports previous research proposing set-shifting as an endophenotype/biomarker or trait characteristic for AN [3]–[5].

Regarding poor learning from feedback, the findings are similar to previous studies which report poor learning in a decision making task [16]–[18] where both patient groups (AN and BN) failed to improve and shift from a disadvantageous strategy (picking risky cards versus safe cards) to an advantageous strategy (picking safe cards with small wins and small amount of losses); again feedback (behavioural and physiological) did not facilitate an improvement in the ED groups' cognitive approach to the task. Both WCST results and decision making studies demonstrate that people with AN and BN have difficulty learning from previous experiences, evidenced by little improvement over time in these neurocognitive tasks. Interestingly, people recovered from AN still were performing less efficiently than HCs. This supports the evidence that individuals recovered from AN have difficulties in differentiating positive and negative feedback [19].

To our knowledge, this study has used the largest available cross-sectional dataset reporting WCST performance administered with the electronic version of the WCST in people with EDs. The strengths of the study are the large sample size, cross-sectional design including currently ill ED groups and recovered groups, as well as an age matched HC comparison group. As highlighted before, experimenter error was reduced to minimum because all studies included here used the computerised version of the WCST. A further strength of the present study is that it reports all outcomes of the WCST in addition to perseverative errors. This has important clinical implications because psychological therapy is focused on learning (unlearning maladaptive behaviours and learning new strategies) and is largely based on feedback. Therefore, understanding the mechanism by which feedback is used by patients is informative.

This study provides an important message about cognitive shifting ability in AN, BN and recovered groups. This line of research is potentially useful to better understand EDs in terms of cognition as well as comorbidity, lifetime diagnosis and personality characteristics and may also help us develop improved treatment approaches. In terms of therapeutic interventions, the recently developed Cognitive Remediation Therapy (CRT) for AN [20]–[21], allows therapists to address flexibility of thinking within therapy sessions and helps individuals develop an awareness about thinking styles and apply this knowledge in their daily lives. Initial feasibility studies [21]–[22] show positive results, suggesting that a rational approach (“cold cognitive route”), raising awareness of thinking styles, might be beneficial for patients with AN. From a recent systematic review [23], it seems that people with AN have good cognitive reserves in terms of higher than average IQ (110). In our dataset it was replicated when we analysed available data on IQ. These strengths can be used as a cognitive resource in therapy. This study highlights that further work and clinical adaptations to support BN patients will be needed. From previous reports, it was not clear whether poor set shifting constitutes part of the neurocognitive signature in BN (because of small scale studies). The present study highlights that more work and clinical adaptations for BN may be needed. Previous reports are less clear about the neurocognitive profile in BN due to the small scale nature of current studies [13].

In the broader context, WCST performance impairment is not specific for ED. Difficulties with cognitive flexibility are reported in almost every psychiatric disorder; however studies of this kind can help us to think about the relative neuropsychological impairments. In general, the WCST allows us to access abstraction ability but clinically it can inform therapists of specific areas of difficulty, e.g. perseverations, conceptualisation, maintaining set or learning through the task over the time. In comparison to brain lesion or schizophrenia patients, performance of people with ED is better (e.g. [24], meta-analysis on schizophrenia reported effect sizes of 1.00 on WCST categories completed and 0.8 on perseverative errors, which are greater than the ED groups performance presented in this study). A meta-analysis of obsessive compulsive disorder patients [25] reported small to medium effect sizes relative to controls performance on WCST categories completed (*d* = 0.23) and perseverative errors (*d* = 0.25), which are lower than the effect sizes reported in the current study.

It is of note that data presented here were merged from different studies available within our department. Therefore, information about medication, illness duration, IQ and subtypes of the ED were not included in the analysis due to missing data, although it should be noted that 70% of patients had IQ measured using the NART and the predicted IQ was 110 (s.d. = 8.9), . In future research, it would be desirable to measure these variables in relation to WCST performance. It was difficult to recruit recovered BN participants and future work would benefit from including this group.

The WCST is the most widely reported neuropsychological measure of executive function and is viewed as an excellent indicator of prefrontal function. There is a growing interest in the diagnostic and treatment implications of cognitive flexibility and reporting all the available outcomes of this most widely disseminated task will be useful to researchers and clinicians alike, working both inside and outside of the ED field.
